# Urine Monocyte Chemoattractant Protein-1 Is an Independent Predictive Factor of Hospital Readmission and Survival in Cirrhosis

**DOI:** 10.1371/journal.pone.0157371

**Published:** 2016-06-30

**Authors:** Isabel Graupera, Elsa Solà, Núria Fabrellas, Rebeca Moreira, Cristina Solé, Patricia Huelin, Gloria de la Prada, Elisa Pose, Xavier Ariza, Alessandro Risso, Sonia Albertos, Manuel Morales-Ruiz, Wladimiro Jiménez, Pere Ginès

**Affiliations:** 1 Liver Unit, Hospital Clínic, University of Barcelona, Barcelona, Spain; 2 Institut d’Investigacions Biomèdiques August Pi I Sunyer (IDIBAPS), Barcelona, Spain; 3 Centro de Investigación Biomédica en Red de Enfermedades Hepáticas y Digestivas (CIBEREHD), Barcelona, Spain; 4 School of Nursing, University of Barcelona, Barcelona, Spain; 5 Biochemistry and Molecular Genetics Department, Hospital Clínic, University of Barcelona, Barcelona, Spain; 6 Department of Physiological Sciences, University of Barcelona, Barcelona, Spain; Hannover Medical School, GERMANY

## Abstract

MCP-1 (monocyte chemoattractant protein-1) is a proinflammatory cytokine involved in chemotaxis of monocytes. In several diseases, such as acute coronary syndromes and heart failure, elevated MCP-1 levels have been associated with poor outcomes. Little is known about MCP-1 in cirrhosis. AIM: To investigate the relationship between MCP-1 and outcome in decompensated cirrhosis. METHODS: Prospective study of 218 patients discharged from hospital after an admission for complications of cirrhosis. Urine and plasma levels of MCP-1 and other urine proinflammatroy biomarkers: osteopontin(OPN), trefoil-factor3 and liver-fatty-acid-binding protein were measured at admission. Urine non-inflammatory mediators cystatin-C, β2microglobulin and albumin were measured as control biomarkers. The relationship between these biomarkers and the 3-month hospital readmission, complications of cirrhosis, and mortality were assessed. RESULTS: 69 patients(32%) had at least one readmission during the 3-month period of follow-up and 30 patients died(14%). Urine MCP-1 and OPN levels, were associated with 3-month probability of readmission (0.85 (0.27–2.1) and 2003 (705–4586) ug/g creat vs 0.47 (0.2–1.1) and 1188 (512–2958) ug/g creat, in patients with and without readmission, respectively; p<0.05; median (IQR)). Furthermore, urine levels of MCP-1 were significantly associated with mortality (1.01 (1–3.6) vs 0.5 (0.2–1.1) μg/g creat, in dead and alive patients at 3 months; p<0.05). Patients with higher levels of urine MCP-1 (above percentile 75^th^) had higher probability of development of hepatic encephalopathy, bacterial infections or AKI. Urine MCP-1 was an independent predictive factor of hospital readmission and combined end-point of readmission or dead at 3 months. Plasma levels of MCP-1 did not correlated with outcomes. CONCLUSION: Urine, but not plasma, MCP-1 levels are associated with hospital readmission, development of complications of cirrhosis, and mortality. These results suggest that in cirrhosis there is an inflammatory response that is associated with poor outcomes.

## Introduction

Increasing evidence suggest that there is a chronic inflammatory reaction in cirrhosis that may play a role in determining patients outcome[1, 2]. This can be an aseptic inflammation, at least in some patients, not related to the presence of detectable infections[3].Several lines of evidence support this hypothesis. High levels of C-reactive protein (CRP) have been found in patients with alcoholic cirrhosis without infections that correlate with liver dysfunction and mortality [4]. In the large cohort of the Canonic study, leukocyte count was an independent predictive factor of 3-month mortality regardless of the presence of detectable infection[5]. Moreover, the presence of SIRS, with and without infection, has been associated with poor outcome in some populations of patients with cirrhosis[6–8].

To gain a further insight in this hypothesis of presence and relevance of systemic inflammation in cirrhosis, we studied monocyte chemoattractant protein-1 (MCP-1) levels in a prospective study in a large series of patients with cirrhosis. MCP-1, also known as chemokine C-C motif ligand 2 (CCL2), is a member of the chemokine family present in a large variety of cell types (endothelial cells, fibroblasts, mononuclear cells) and is one of the most highly expressed chemokines during inflammation[9, 10]. MCP-1 acts as a potent chemotactic factor that regulates recruitment of monocytes to the inflammation site or tissue injury[11]. The role of systemic MCP-1 has been evaluated in several chronic inflammatory states, and has been found to be associated with poor outcomes[12–16]. Plasma levels of MCP-1 proved to be an independent prognostic marker in acute coronary syndrome and also a predictor of all cause of mortality in patients with heart failure[12, 13]. Accumulating evidence from experimental studies shows that MCP-1 compromises the integrity of the blood-brain barrier and modulates the progression of various diseases in central nervous system disorders[14]. Evidence from human studies also shows that there is immunochemical expression of MCP-1 in active lesions of multiple sclerosis in autopsied brains[10]. Moreover, MCP-1 modulates the inflammatory response in renal diseases and increases inflammatory molecules on mesangial and tubular cells[15]. Finally, the inhibition of MCP-1 has been shown to ameliorate a variety of inflammatory renal diseases, including diabetic nephropathy [16].

Hepatic gene expression of MCP-1 is increased in patients with chronic liver diseases[17]. MCP1 appears to play an important role in liver inflammation and fibrogenesis, driving macrophage infiltration by binding CCR2 receptor[18–21]. However, compared to other chronic diseases, little is known about systemic MCP-1 and cirrhosis. Increased levels of plasma MCP-1 have been found in patients after large-volume paracentesis and also in patients with spontaneous bacterial peritonitis and are associated with derangement of circulatory function and impaired survival, respectively [22, 23]. Moreover, we have previously shown that increased urinary levels of MCP-1 are associated with the presence of acute-on-chronic liver failure (ACLF)[24].

On this background, the current study was aimed at assessing the relationship between MCP-1 and prognosis in decompensated cirrhosis. We analyzed urine and plasma levels of MCP-1 in a prospective series of consecutive patients hospitalized for complications of cirrhosis and evaluated the probability of hospital readmission, development of complications, and survival at 3 months. Our results show that urine but not plasma MCP-1 levels are associated with hospital readmission, development of complications and poor survival in patients with decompensated cirrhosis

## Methods

### Study population

This was a prospective study that included 218 consecutive patients with cirrhosis hospitalized for management of an acute decompensation of the disease at the Liver Unit of Hospital Clinic of Barcelona within a 2-year period. Patients were excluded from the study if: (1) had chronic kidney disease treated with hemodialysis before admission, (2) had previous liver or kidney transplantation, (3) had hepatocellular carcinoma beyond Milan criteria, (4) had advanced chronic respiratory or heart disease (NYHA III and IV), and (5) had severe extrahepatic diseases with poor short-term prognosis. Out of the 265 patients eligible during study period, 29 patients died and 5 were transplanted during hospitalization and 13 patients did not have urine sample. Finally, we included 218 patients to assess the role of urine and plasma MCP-1 and other urinary inflammatory mediators in predicting patient’s outcome after hospital discharge.

### Study design

Demographical, clinical and analytical data were collected from all patients at study inclusion. In addition, 10 mL of urine were obtained at inclusion to measure urine MCP-1 levels. In addition to MCP-1 we also measured other urinary inflammatory mediators, including osteopontin (OPN), liver-fatty-acid-binding protein (LFABP), and trefoil-factor 3 (TFF3). These biomarkers were selected from a number of biomarkers on the basis of their association with patients outcomes in a previous study from our group [24]. We also measured urinary β2-microglobulin (β2M), Cystatin-C (Cys-C), and albumin levels as control non-inflammatory mediators. At the same time of urine collection, 10 mL of blood were obtained to measure plasma levels of MCP-1.

After discharge, patients were followed-up in the outpatient clinic for at least 3 months and all complications of cirrhosis occurring during this period were recorded prospectively. All patients gave written informed consent and the study was approved by the Institutional Review Board Comitè Ètic d’Investigació Clínica del Hospital Clínic de Barcelona.

Urine samples were collected at 9 a.m. and centrifuged at 1000rpm for 10 minutes within the first 4 hours and stored at -80° until use. MCP-1 and L-FABP were measured by Human CCL2/MCP1 Quantikine ELISA Kit (R&D Systems) and Human L-FABP ELISA (Hycult Biotech) respectively. Albumin measurement was performed by ADVIA ChemistryMicroalbumin_2 immunoturbidimetry method, enhanced with polyethylene glycol, (Siemens Healthcare Diagnostics, Deerfield, IL). β2-microglobulin, cystatin C, osteopontin, and TFF- 3 were measured with RBM Human Kidney Toxicity Panel 2 according to the manufacturer protocol (Bio-Rad Laboratories, Hercules, CA). Briefly, urine samples were centrifuged at 500 x g for 5 min and diluted 1:50 for the assays. After blockade of nonspecific binding sites, 30 microliters of standards, controls or diluted samples were added to 96-well plates and incubated with 10 mL of fluorescently dyed magnetic microspheres covalently coupled to specific antibodies for the desired biomarkers. Next, the plates were incubated for 1 h at room temperature and washed three times with wash buffer. Afterwards, 40 mL of biotinylated detection antibody was added to the wells and the plates were incubated for an additional hour at room temperature. The final detection complex was formed with the addition of streptavidin-phycoerythrin conjugated. The median relative fluorescence units from the antibody reactions were acquired using a Luminex 200 analyzer (Luminex, Austin, TX, USA) and the Bio-Plex Manager Software (v. 6.0; Bio-Rad Laboratories, Hercules, CA). The urinary biomarkers were indexed to urinary creatinine for all analyses to adjust for variability in urine concentration.

Plasma samples were obtained at 9.a.m and centrifuged at 2000 rpm for 10 minutes and the supernatant was stored at -80°C until analysis. MCP-1 was measured by Human CCL2/MCP-1 Quantikine ELISA (R&D Systems).

We examined the relationship between biomarkers and probability of 3-month hospital readmission, 3-month mortality and a combined end-point of 3-month hospital readmission and mortality. We also analyzed the relationship between these biomarkers and complications of cirrhosis developing during follow-up period. Complications of cirrhosis were defined as development of new-onset ascites or worsening of existing ascites (defined by increase of diuretic therapy or need for large-volume paracentesis), hepatic encephalopathy (as assessed clinically), bacterial infections, gastrointestinal bleeding or acute kidney injuri (AKI). The diagnosis of ascites, hepatic encephalopathy, bacterial infections and GI bleeding was made with criteria reported elsewhere[25–28]. AKI was diagnosed according to AKIN criteria[27].

### Statistical analysis

Continuous variables are presented as mean ± standard deviation or median (interquartile range), and categorical variables are presented as number and percentage. We used independent-sample t-tests to compare continuous variables and chi-square tests to compare categorical variables. Kaplan-Maier method was used to estimate the frequency of readmission, death and complications of cirrhosis during 3-month follow-up. The log-rank test was used to compare outcomes between patients with MCP-1 levels above or equal to or below the 75^th^ percentile (MCP-1≥p75 or MCP-1<p75). The selection of a dichotomous threshold at 75^th^ percentile was retrospectively defined. Finally, a logistic regression model was created to identify the best independent predictive factors of readmission and survival. All variables that were predictive of outcomes at univarate analysis were included in the multivariate model. Urine MCP-1 was introduced in the model as a dichotomous variable according to percentile 75^th^ (MCP-1≥p75 or MCP-1<p75). Variables with skewed distribution were log-transformed before being included in the multivariate analysis model. All statistical analyses were performed using SPSS 20.0 software. The significance level for all statistical tests was set at 0.05 two-tailed.

## Results

### Characteristics of the patient population

Demographic and clinical characteristics of the cohort are shown in Table 1. Patients had moderate to severe impairment of liver function as reflected by high serum bilirubin, low serum albumin, and mean MELD score of 16.

**Table 1:** Demographic, clinical and laboratory data of the 218 patients included in the study.

VARIABLE		
Age (years)	60 ± 12	(21–84)
Gender, male	141 (65%)	
Alcoholic cirrhosis	101(46%)	
Cause of admission		
Ascites	53 (24%)	
Hepatic encephalopathy	33 (15%)	
Non-SBP infections	36 (16%)	
SBP	13 (6%)	
GI bleeding	21 (9%)	
Other	62 (28%)	
Serum bilirubin (mg/dL)	5 ± 6	(0.3–32)
Serum albumin (g/L)	27 ± 5	(17–44)
INR	1.7±0.5	(0.9–4.4)
Serum creatinine (mg/dL)	1±0.6	(0.4–4)
Serum sodium (mEq/L)	135±6	(119–148)
Leukocyte count (10^9^ cells/L)	6.2±4	(0.6–39)
CRP (mg/dL)	3.8 ±10	(0.02–129)
AKI*	50 (23%)	
MELD score	16±7	(4–38)
Child Pugh:		
Score	10 ± 2	
Class A/B/C (%)	10% / 48% / 42%	
Plasma MCP-1 (pg/mL)	248	(197–331)
Urine biomarkers:		
MCP-1 (μg/g creat)	0.54	(0.2–1.4)
OPN (μg/g creat)	1466	(532–3461)
TFF-3 (μg/g creat)	1032	(448–3046)
LFABP (μg/g creat)	20	(8–46)
Albumin (mg/g creat)	7.5	(2–27)
Cys-C (μg/g creat)	36.2	(14–81)
β2M (μg/g creat)	90	(25–288)

Data are expressed as mean±SD and ranges or number and percentages. Biomarkers are expressed as median (IQR). MCP-1: Monocyte chemoattractant protein 1, OPN: osteopontin, TFF3: Trefoil-factor3, LFABP: Liver-fatty-acid-binding protein, Cys-C: cystatin C, β2M: β2microglobulin. Values of urine biomarkers was measured in a subgroup of 6 healthy subjects (4 male, mean age of 59 ±8) and are as follows: MCP-1 0.1 (0.04–0.2) μg/g creat, Osteopontin 1416 (900–2025) μg/g creat, TFF-3 678 (466–951) μg/g creat, Albumin 1 (0–2) mg/g creat, Cystatin C 32 (8–57) μg/g creat, β2-microglobulin 95 (9–132) μg/g creat [18], LFAB-P 0.22±0.08 (mean ±SD). Levels of plasma MCP-1 were measured in a subgroup of 13 healthy subjects: MCP-1 plasma: 210 (163–251) pg/mL *Etiologies of AKI were: pre-renal n = 20 (40%); Hepatorenal syndrome n = 14 (28%); Acute tubular necrosis n = 7 (14%), Nephrotoxic drugs n = 5 (10%); Other n = 4 (8%)

### Biomarker levels and relationship with inflammatory parameters and liver and kidney tests

Urine levels of biomarkers are presented in Table 1. In the whole series, urinary levels of MCP-1 and albumin were increased compared to those of healthy subjects (MCP-1:0.54(0.2–1.4) vs 0.1(0.3–3.7) μg/g creat and albumin: 7.5(2–27) vs 1.3(0–2.1) mg/gcreat, both p<0.05). The remaining urinary biomarkers were not increased with respect to normal values. We analysed the relationship between urinary biomarkers and parameters of inflammation and liver and kidney function tests. Inflammatory mediators (MCP-1, OPN, TFF-3 and L-FABP) had a direct yet poor correlation with C-reactive protein (CRP) (r = 0.1, r = 0.16, r = 0.14 and r = 0.17; p<0.05), but not with leukocyte count (except for L-FABP; r = 0.16; p<0.001). Urine MCP-1 had a negative correlation with albumin (r = - 0.14; p<0.001) and positive correlation with INR and MELD score (r = 0.16 and r = 0.14, both p<0.001). There was a poor, but statistically significant, direct correlation between urine MCP1 and TFF-3 with serum creatinine levels (r = 0.1 and r = 0.14, respectively; p<0.05). Moreover, there was a negative correlation between urine Cys-C and β2M and serum creatinine levels (S1 Fig). With respect to plasma MCP-1, patients with decompensated cirrhosis had higher levels compared to healthy subjects (248 (196–331) vs 210 (163–254) pg/mL, p = 0.28). There was a significant direct correlation between plasma MCP-1 levels and serum creatinine (r = 0.15; p<0.05. S1 Fig), bilirubin (r = 0.13; p<0.05), and MELD score (r = 0.18; p<0.001). By contrast, no correlation was found between plasma MCP-1 levels and other parameters of inflammation such as CRP levels or leukocyte count.

Finally, we compared plasma and urine levels of MCP-1 and there was a poor direct correlation between them (r = 0.13, p = 0.04; S1 Fig).

### Biomarker levels and relationship with complications of cirrhosis at hospital admission

Patients with ascites and HE at admission had significantly higher levels of urinary MCP-1 and OPN compared to those of patients without these complications (0.73 (0.27–1.59) and 1688 (655–4240) vs 0.38 (0.15–0.74) and 1068 (283–2505) ug/g creat, p <0.001 for patients with ascites; 0.79 (0.35–2.01) and 1990 (594–4757) vs 0.48 (0.20–1.08) and 1216 (508–3008), ug/g creat, p<0.05 for patients with hepatic encephalopathy). By contrast, there were no significant differences in urinary biomarker levels when patients with and without bacterial infections were compared. Because of the statistically significant correlation between urine MCP-1 and serum creatinine, we analyzed whether levels of urine MCP-1 and other biomarkers could be related to kidney dysfunction. Patients were categorized according to quartiles of serum creatinine and the levels of each biomarker compared among the different quartiles. Urine TFF-3 levels increased significantly in parallel to quartiles of serum creatinine. By contrast, urine Cys-C and β2M correlated inversely with serum creatinine levels (S1 Table). We also compared levels of all urine biomarkers between patients with and without AKI at admission, and urine TFF-3 was the only urine biomarker significantly increased in patients with AKI compared to patients without AKI (S2 Table).

In contrast to the relationship between urine MCP-1 levels and some of the complications of cirrhosis, there was no relationship between plasma MCP-1 and presence of complications of cirrhosis at admission to hospital, except for higher plasma levels of MCP-1 in patients with AKI compared to those of patients without AKI (S2 Table).

### Biomarker levels and relationship with patient’s outcomes

#### Hospital readmission and mortality

At the end of 3-month follow period, 69 patients (32%) had had at least one readmission to hospital for complications of cirrhosis. The most common causes of readmission were bacterial infections and HE, which accounted for almost half of all readmissions (17 patients and 13 patients, respectively). During the 3-month follow-up period, 30 patients died (14%), 10 patients were transplanted, and 4 were lost to follow-up. The main cause of death was acute-on-chronic liver failure, which occurred in 21 patients (70%). Out of the total 218 patients, 78 patients (36%) developed the composite end-point of 3-month readmission or mortality.

Table 2 shows the univariate analysis of variables related to hospital readmission during the 3-month period. Besides liver tests and prognostic scores, urine MCP-1 and OPN levels were significantly higher in patients who had at least one readmission to hospital compared to those without readmissions. No other biomarkers were associated with hospital readmission. Of note, plasma MCP-1 levels were similar in patients with readmission compared to those without.

**Table 2:** Univariate analysis of variables obtained at hospital admission associated with 3-month readmission during follow up.

	No readmission (n = 149)	Readmission (n = 69)	p value
Age (years)	60±12	60±11	0.7
Sex (male)	98 (66%)	44 (63%)	0.6
Alcoholic Cirrhosis	70 (47%)	31 (45%)	0.7
Ascites	92 (62%)	54(78%)	0.02
Hepatic encephalopathy	41 (27%)	21(30%)	0.6
AKI	40 (33%)	22 (38%)	0.6
Non-SBP infection	25 (17%)	11 (16%)	1.0
SBP infection	10 (7%)	3 (4%)	0.7
Serum bilirubin (mg/dL)	4.2 ± 6.3	6.1 ± 7.2	0.05
Serum albumin (g/L)	27 ± 5	26 ± 4	0.3
INR	1.6 ± 0.5	1.8 ± 0.5	0.002
Serum creatinine (mg/dL)	1.0 ± 0.6	1.1 ± 0.6	0.6
Serum sodium (mEq/L)	135 ± 5	135 ± 4	0.7
CRP (mg/dL)	2 (1–3.7)	1.9 (0.6–4)	0.4
Leukocyte count (x10^9^/L)	6.2 ± 4.5	6.4 ± 4.3	0.8
MELD score	15 ± 7	18 ± 7	0.003
Child-Pugh:			
Score	9 ± 2	10 ± 2	0.002
Class A/B/C	12% / 54% / 34%	7% /36% / 57%	0.013
Plasma MCP-1 (pg/mL)	241 (193–341)	266 (200–331)	0.4
Urine Biomarkers:			
MCP-1 (μg/g creat)	0.47 (0.2–1.07)	0.82 (0.3–2.0)	0.01
OPN (μg/g creat)	1188 (512–2958)	2003 (705–4586)	0.049
TFF3 (μg/g creat)	1582 (413–3894)	938 (462–2538)	0.1
LFABP (μg/g creat)	24 (11–70)	18 (7–41)	0.2
Albumin (mg/g creat)	12 (3–34)	7 (2–24)	0.2
β2M (μg/g creat)	83 (19–434)	94 (28–241)	0.6
Cys-C (μg/g creat)	34 (12–105)	39 (15–79)	0.7

Data are expressed as mean±SD, median (interquartile range) or number and percentages.CRP (C reactive protein), AKI (Acute kidney injury), SBP (spontaneous bacterial peritonitis); MCP-1: Monocyte chemoattractant protein 1, OPN: osteopontin, TFF3: Trefoil-factor3, LFABP: Liver-fatty-acid-binding protein, Cys-C: cystatin C, β2M: β2microglobulin.

Table 3 shows the univariate analysis of variables related to 3-month mortality. Besides clinical decompensations, liver tests, and prognostic scores, urinary MCP-1 and OPN levels were associated with mortality. No other urine biomarkers or plasma MCP-1 were associated with mortality.

**Table 3:** Univariate analysis of variables obtained at hospital admission associated with 3-month mortality during follow-up.

	Alive (n = 188)	Dead (n = 30)	p value
Age (years)	60 ±11	60 ±12	0.9
Sex (male)	123 (65%)	18 (60%)	0.6
Alcoholic Cirrhosis, n (%)	86 (46%)	15 (50%)	0.8
Ascites	125 (66%)	21 (70%)	0.8
Hepatic encephalopathy	43 (23%)	19 (63%)	<0.001
AKI*	48 (21%)	14 (58%)	0.02
Non-SBP infection	35 (18%)	1 (3%)	0.04
SBP infection	10 (5%)	3 (10%)	0.4
Serum bilirubin (mg/dL)	4.2 ±5	8.5 ± 8.6	0.01
Serum albumin (g/L)	27 ± 5	26 ± 5	0.5
INR	1.6± 0.5	2 ± 0.6	0.003
Serum creatinine (mg/dL)	1 ± 0.5	1.4 ± 0.8	0.01
Serum sodium (mEq/L)	135 ± 5	134 ± 3	0.3
Leukocyte count (x10^9^/L)	5.8 ±4	8.6 ± 5	0.001
MELD score	14 ± 7	22 ± 7	<0.001
Child- Pugh:			
Score	9 ± 2	11 ± 2	<0.001
Class A/B/C	11% / 53% / 36%	7% / 18% / 75%	0.001
Plasma MCP1 (pg/mL)	243 (195–331)	295 (226–338)	0.18
Urine Biomarkers:			
MCP-1 (μg/g creat)	0.5 (0.2–1.1)	1.01 (1–3.6)	0.02
OPN (μg/g creat)	1315 (504–3269)	2324 (767–5497)	0.05
TFF3 (μg/g creat)	1012 (453–2772)	1929 (365–3895)	0.2
LFABP (μg/g creat)	18 (8–42)	21 (11–106)	0.1
Albumin (mg/g creat)	7 (2–27)	16 (4–74)	0.07
β2M (μg/g creat)	91 (27–272)	67 (10–379)	0.4
Cys-C (μg/g creat)	36 (14–78)	34 (9–188)	0.6

Data are expressed as mean±SD, median (interquartile range) or number and percentages.CRP (C reactive protein),

*AKI (Acute kidney injury), SBP (spontaneous bacterial peritonitis); MCP-1: Monocyte chemoattractant protein 1, OPN: osteopontin, TFF3: Trefoil-factor3, LFABP: Liver-fatty-acid-binding protein, Cys-C: cystatin C, β2M: β2microglobulin.

In multivariate analysis, the only independent predictive factors of hospital readmission and combined end-point of hospital readmission or death were urine MCP-1 and MELD score (Table 4).

**Table 4:** Multivariate models of 3-month hospital readmission and combined end-point of 3-month hospital readmission or death. Model 1. 3-month hospital readmission. Model 2. Combined end-point of 3-month hospital readmission or death.

**Model 1**	**HR**	**95% IC**	**p-value**
Urine MCP-1≥p75	2.1	1.06–4.36	0.03
MELD	1.07	1.02–1.1	0.02
**Model 2**	**HR**	**95% IC**	**p-value**
Urine MCP-1≥p75	2.4	1.19–4.88	0.01
MELD	1.08	1.03–1.1	0.001

Model 1: Variables included in the model: MELD score, CHILD score, urine MCP-1≥p 75^th^; Model 2: Variables included in the model: MELD score, CHILD score, urine MCP-1≥p75

Because urine MCP-1 was the only biomarker independently associated with readmission and the combined end point of 3-month hospital readmission or mortality, we focused our analyses on urine MCP-1 and its relationship with different outcomes. The characteristics of patients according to quartiles of urine MCP-1 are shown in Table 5. Patients with urine MCP-1 in the fourth quartile (MCP-1>p75) had more advanced liver disease, as indicated by greater frequency of previous complications of cirrhosis (ascites, HE or infection), compared to those in the other quartile groups.

**Table 5:** Baseline characteristics of patients categorized according to quartiles of urine MCP-1.

	Q1 (n = 53)	Q2 (n = 55)	Q3 (n = 56)	Q4 (n = 54)	p
MCP-1 range (ug/g creat)	0.01–0.22	0.23–0.54	0.55–1.43	1.44–26	—
Age	62 ±12	60 ±12	59 ±11	60 ±11	0.5
Male sex, n(%)	33 (58%)	32 (60%)	37 (69%)	39 (73%)	0.3
Diabetes Mellitus, n(%)	12 (21%)	14 (26%)	13 (25%)	14 (26%)	0.9
Arterial Hypertension	12 (21%)	10 (19%)	5 (9%)	10 (19%)	0.4
Etiology cirrhosis:					
Alcohol	25 (44%)	25 (47%)	26 (49%)	25 (47%)	0.8
Hepatitis C	25 (44%)	26 (49%)	22 (42%)	23 (44%)	
Previous decompensation:					
Previous ascites, n(%)	33 (58%)	28 (53%)	43 (81%)	41 (77%)	0.002
Previous HE, n(%)	10 (17%)	16 (30%)	11 (21%)	24 (45%)	0.02
Previous infections, n(%)	6 (10%)	5 (9%)	8 (15%)	20 (37%)	<0.001
Decompensation at admission:					
Ascites	33 (58%)	28 (53%)	43 (82%)	41 (76%)	0.003
Hepatic encephalopathy	10 (17%)	16 (30%)	12 (23%)	25 (46%)	0.03
Infection non-SBP	10 (17%)	7 (13%)	9 (17%)	10 (18%)	0.8
SBP	5 (9%)	1 (2%)	4 (7.5%)	3 (6%)	0.4
AKI	10 (20%)	7 (15%)	8(19%)	15 (34%)	0.1
SIRS	24 (51%)	19 (35%)	17 (32%)	22 (40%)	0.5
Serum bilirubin (mg/dL)	4 ± 6	5 ± 7	5 ± 7	4 ± 6	0.7
Serum albumin (g/L)	28 ± 5	26 ± 4	26 ± 5	26 ± 5	0.05
INR	1.5 ± 0.5	1.6 ± 0.4	1.8 ± 0.6	1.8 ± 0.6	0.02
Serum creatinine (mg/dL)	1 ± 0.7	1 ± 0.5	1 ± 0.6	1.1 ± 0.6	0.7
Serum sodium (mEq/L)	135 ± 6	136 ± 4	134 ± 4	134 ± 4	0.2
Leukocyte count (10^9^ cells/L)	5 ± 3	7 ± 5	6 ± 6	7 ± 4	0.2
CRP (mg/dL)	2.7 ± 4	3 ± 3	3.6 ± 4	3.2 ± 3	0.6
MELD score	14 ± 8	14 ± 6	17 ± 8	17 ± 8	0.05
Child-Pugh score	9 ± 2	9 ± 2	10 ± 2	10 ± 2	0.003

Data are expressed as mean±SD and ranges or number and percentages. SIRS: systemic inflammatory response; CRP: C- reactive protein. AKI at admission was defined according to AKIN criteria (21)

**Fig 1:**
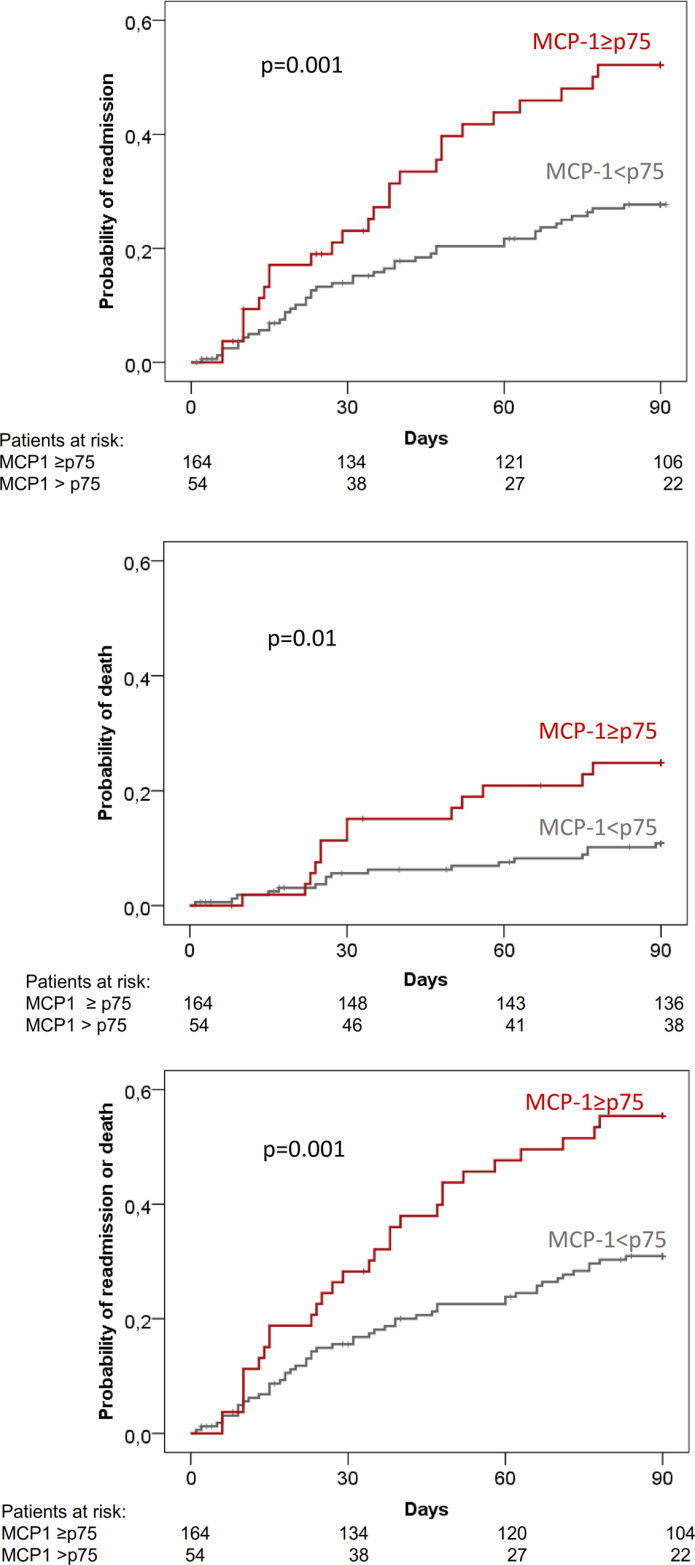
3-month probability of hospital readmission, death and combined end-point of hospital readmission or death in all patients categorized according to fourth quartile of urine MCP-1 levels.

There was a statistically significant difference of outcomes (readmission, death, composite of death or readmission and complications) across the quartile groups of MCP-1 (Table 6). However, a closer look at this relationship showed that outcome rates were quite similar in the first three quartiles of MCP-1 levels, and increased markedly in the fourth quartile group (Table 5). In fact, when compared with the first three quartiles, patients in the fourth quartile had significantly higher probability of readmission (48% vs 26%; p = 0.001), death (24% vs 10%); p = 0.01), and composite end-point of readmission and death (54% vs 30%; p = 0.001) (Fig 1).

**Table 6:** Outcomes according to quartiles of urine MCP-1.

	Q1	Q2	Q3	Q4	p
	(n = 53)	(n = 55)	(n = 56)	(n = 54)	**value**
MCP-1 range (μg/g creat)	0.01–0.22	0.23–0.54	0.55–1.43	1.44–26	—
3-month readmission	12 (23%)	14 (25%)	17 (32%)	26 (48%)	0.02
3-month mortality	5 (9%)	6 (11%)	6 (9%)	13 (24%)	0.09
3-month readmission or death	15 (28%)	14 (25%)	20 (36%)	29 (53%)	0.01

#### Complications of cirrhosis during follow-up

**Fig 2:**
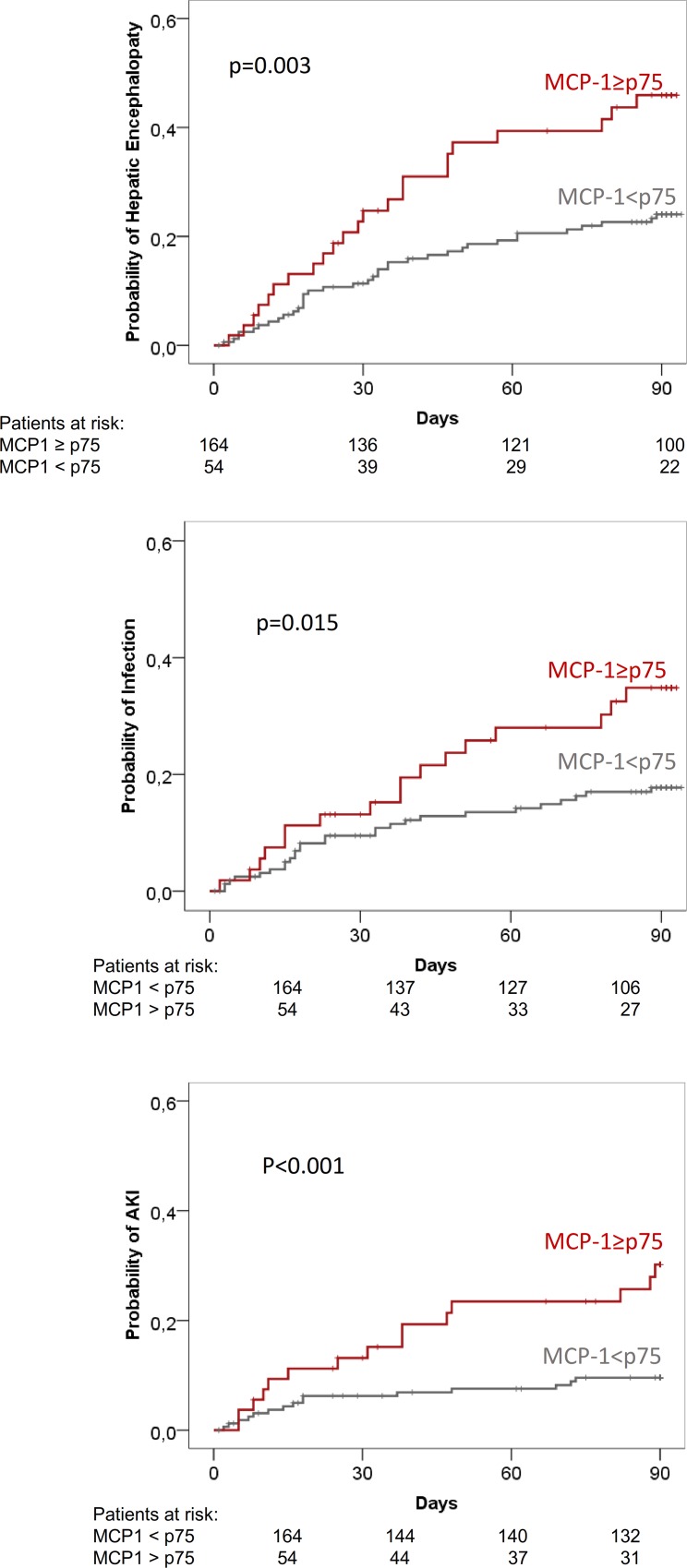
3-month probability of EH, bacterial infections and AKI in all patients categorized according to fourth quartile of urine MCP-1 levels.

During the 3-month follow-up period, 100 of the 218 patients (46%) developed a total of 181 complications of cirrhosis (Table 7). The most common complication was HE followed by bacterial infections. Urine MCP-1 levels were significantly higher in patients who subsequently developed any complication during follow-up compared to those of patients who did not This was true for all individual complications except for ascites and gastrointestinal bleeding (Table 7). In contrast to differences observed in urine MCP-1 levels, there were no significant differences in plasma MCP-1 between patients who did and did not develop complications (Table 7). Fig 2 shows the 3-month probability of development of HE, bacterial infections, and AKI in the whole population of patients divided into two groups: patients within the first three quartiles (MCP1<p75) and patients within the fourth quartile of urine MCP-1 (MCP-1≥p75). Patients in the fourth quartile of urine MCP-1 levels had higher probability of developing the later complications compared to patients in the first three quartiles of MCP-1.

**Table 7:** Urine and plasma MCP-1 levels according to development of complications of cirrhosis during follow up.

COMPLICATIONS OF CIRRHOSIS	Urine MCP-1 (μg/g creat)			Plasma MCP-1 (pg/mL)		
	YES	NO	p	YES	NO	p
Any complication (n = 100)	0.74	0.47	0.02	252	246	0.4
	(0.23–1.96)	(0.21–1.05)		(205–313)	(181–3419)	
Hepatic encephalopath(n = 60)	0.93	0.48	0.01	256	246	0.5
	(0.32–2.18)	(0.2–1.12)		(211–313)	(195–332)	
All infections (n = 44)	0.84	0.51	0.07	264	243	0.6
	(0.26–3.15)	(0.21–1.14)		(195–316)	(196–332)	
SBP (n = 6)	4.28	0.51	<0.001	196	249	0.2
	(3.75–7.42)	(0.21–1.25)		(111–317)	(198–331)	
Ascites (n = 34)	0.73	0.53	0.5	224	251	0.6
	(0.2–2.09)	(0.22–1.26)		(197–314)	(196–331)	
All AKI (n = 30)	1.43	0.49	0.01	273	246	0.6
	(0.41–3.72)	(0.21–1.12)		(190–362)	(198–331)	
HRS (n = 8)	2.8	0.51	0.005	281	246	0.7
	(1.4–10.5)	(0.21–1.24)		(183–362)	(194–331)	
GI bleeding (n = 13)	1.24	0.54	0.2	200	250	0.07
	(0.21–4.02)	(0.22–1.37)		(170–275)	(199–331)	

Data are expressed as median (interquartile range). Numbers in brackets after each complication represent the number of patients who developed each complication during the 3-month follow-up period. SBP: spontaneous bacterial peritonitis. HRS: hepatorenal syndrome. GI bleeding: gastrointestinal bleeding.

Of the 218 patients included in the study, 128 (58%) were treated with lactulose and 49 (22%) with rifaximin after hospital discharge for prevention of recurrent HE. The probability of development of HE was higher in patients in the fourth quartile of urine MCP-1 in both subgroups of patients, those treated with lactulose and those with rifaximin, compared to the remaining patients, yet the difference did not reach statistical significance (48% vs 32%, p = 0.08, in patients treated with lactulose; and 69% vs 40%, p = 0.05 in patients treated with rifaximin).

Because of the direct correlation between urine MCP-1 and serum creatinine, we repeated all the analysis excluding patients with AKI at inclusion and found similar results. Urine MCP-1 levels were higher in patients who had hospital readmission (0.82 (0.01–5.7) vs 0.46 (0.01–19) μg/g creat; p = 0.02) or died within 3-months (1.24 (0.36–3.3) vs 0.49 (0.2–1.06) μg/g creat; p = 0.03). Patients in the fourth quartile had higher probability of readmission or death during the 3 month period of follow-up (S2 Fig). Urine levels MCP-1 were also a predictive factor of combined end-point of hospital readmission or death in multivariate analysis (data not shown).

## Discussion

The main finding of the current study is that urine MCP-1 levels are associated with hospital readmission and mortality within 3-months. MCP-1 is a potent chemoattractant protein highly expressed in inflammatory states and higher levels have been associted with poor outcomes in several chronic diseases. To our knowledge this is the first time that urinary levels of MCP-1 have been reported as associated with poor outcome in cirrhosis.

Patients with advanced cirrhosis may have increased blood leukocyte count, CRP levels, pro-inflammatory cytokines, and systemic oxidative stress in the absence of infections, which supports the hypothesis that there is a ‘sterile systemic inflammation’ in cirrhosis that may play a role in the progression of the disease[1, 3–5, 8]. MCP-1 is one of the key chemokines that participate in the recruitment of inflammatory cells and is highly expressed under inflamatory conditions. The increased urine and plasma MCP-1 levels found in patients with decompensated cirrhosis in the current study support the hypothesis of the existence of a systemic inflammation in patients with advanced liver disease. In our series, patients with higher levels of urine MCP-1 had higher probability of hospital readmission or death during follow-up compared with patients with lower levels, suggesting that systemic inflammation in cirrhosis is associated with poor outcomes.

An intriguing finding of our study was that whereas hospital readmissions, death, and complications of cirrhosis were highly correlated with urine MCP-1 levels, no correlation was found between these outcomes and plasma MCP-1 levels. The reason(s) for these apparently inconsistent findings remains speculative. With the design of the current study, it is not possible to know whether the levels of MCP-1 found in the urine have a systemic origin, a kidney origin or a combination of both. The systemic production of MCP-1 may theoretically be increased as a result of the inflammatory syndrome characteristic of advanced cirrhosis, with stimulation of the innate immune response[1, 29]. The MCP-1 originated in different organs could then be in part eliminated through the kidneys by glomerular filtration because of the low molecular size of the peptide (13kDa). To our knowledge, only few studies have been reported assessing MCP-1 plasma levels in cirrhosis. In the first study, plasma levels of MCP-1 increased in association with changes in circulatory function after large-volume paracentesis in a very small number of patients, suggesting monocyte activation in the context of large-volume paracentesis induced circulatory dysfunction[22]. In a second study, plasma and ascitic levels of MCP-1 were increased in patients with SBP compared to patients with cirrhosis without SBP, and higher levels were associated with poor survival[23]. Finally, increased plasma levels of MCP-1 were found in patients with cirrhosis and superimposed alcoholic hepatitis compared to patients with cirrhosis without alcoholic hepatitis and healthy subjects. Although plasma levels of MCP-1 correlated with disease severity, there was no relationship between plasma levels and survival[30]. On the other hand, there is evidence that MCP-1 is produced in the kidneys and the renal production of MCP-1 may be increased in a number of conditions, including diabetic nephropathy and systemic lupus erithematosus [16]. In this regard, it is interesting to note that there is an increased expression of toll-like receptor 4 in the renal tubules in cirrhosis^36-37^. Therefore, it could be speculated that the increased endotoxin levels, characteristic of advanced cirrhosis, may enhance the local production of MCP-1 in the kidneys via stimulation of toll-like receptors. The poor correlation observed in our study between plasma and urine MCP-1 levels supports the existence of an increased renal production of MCP-1 in cirrhosis. Nonetheless, regardless of the origin, the results of our study clearly indicate that urine but not plasma MCP-1 levels correlate with outcomes in patients with cirrhosis discharged from hospital after management of an acute decompensation of cirrhosis.

An interesting finding that deserves a comment is the association between MCP-1 levels and the probability of developing complications during follow-up. In our series, urine MCP-1 levels were associated with an increased risk of HE. The pathophysiology of hepatic encephalopathy is not completely understood but increasing evidence indicates that there is a synergistic relationship between inflammation and ammonia in the pathogenesis of HE[31]. Inflammation can arise directly within the brain itself but may also originate in the peripheral circulation and exert effects on the brain indirectly, via the release of pro-inflammatory mediators[32]. We hypothesize that besides being a biomarker of systemic inflammation, MCP-1 may also alter the integrity of the blood brain barrier, as it does in neurologic disorders, decreasing the threshold for HE. In our series, urine MCP-1 levels correlated with the development of bacterial infections, particularly SBP during follow-up. Our results are in keeping with previous studies, where increased levels of MCP-1 were found in patients with SBP compared to patients with ascites without SBP^23^. Finally, urine MCP-1 was also associated with the development of AKI. The presence of severe inflammatory response has been previously associated with development of HRS, a characteristic type of AKI in cirrhosis[33–35]. Findings derived from human and experimental studies have shown that inflammation and infection in cirrhosis lead to tubular damage and renal injury via up-regulation of renal tubular TLR4[36, 37]. It could therefore be speculated that urine MCP-1 could be a marker of systemic inflammation secondary to systemic and local production, and could be an early marker of kidney inflammation and anticipate the development of AKI in patients with cirrhosis.

The current study was performed in patients with cirrhosis hospitalized for management of an acute decompensation. The exclusion of the patients who died during hospitalization is an intrinsic bias of the study. In fact, we excluded patients who died during hospitalization because the aim of the study was to analyse outcomes after discharge. Nevertheless, we excluded patients with poorer prognosis and despite of this MCP-1 was a biomarker of prognosis in the remaining population. Due to the design of the study, we do not know if levels of plasma or urine MCP-1 change over time and if these changes could predict more accurately outcomes in these patients compared to a simple measurement, but this could be addressed in future studies. MCP-1 has been reported as potential biomarker of kidney injury because its expression is increased in kidney cells in experimental conditions of AKI[38]. Therefore, we addressed the question of whether increased MCP-1 levels could be related to the existence of AKI. Nonetheless, there was no statistical significant difference in MCP-1 levels between patients with and without AKI. Moreover, MCP-1 levels did not show a progressive increase in patients categorized according to quartiles of serum creatinine. Furthermore, MCP-1 levels were still an independent predictive factor of poor outcome after exclusion of patients with AKI. Therefore, it appears that increased urine levels of MCP-1 found in the current series of patients were not related to the presence of AKI.

In summary, the results of the current study indicate that urine but not plasma MCP-1 levels are associated with 3-month hospital readmission and mortality. These results suggest that in cirrhosis there is an inflammatory response that is associated with poor prognosis.

## Supporting Information

S1 Fig Correlations between biomarkers and serum creatinine and between urine and plasma MCP-1 levels. Figure A shows correlation between urine MCP-1 and serum creatinine. Figure B shows correlation between urine TTF-3 and serum creatinine. Figure C shows correlation between urine Cys-C and serum creatinine. Figure D shows correlation between urine β2M and serum creatinine. Figure E shows correlation between plasma MCP-1 and serum creatinine. Figure F shows correlation between urine MCP-1 and plasma MCP-1 levels. MCP-1: Monocyte chemoattractant protein 1, OPN: osteopontin, TFF3: Trefoil-factor3, LFABP: Liver-fatty-acid-binding protein, Cys-C: cystatin C, β2M: β2microglobulin
(PPTX)

S2 Fig 3-month probability of hospital readmission, mortality and combined end-point of hospital readmission or death in patients without AKI at index hospitalization categorized according to fourth quartile of urine MCP-1 levels. (PPTX)

S1 Table Supplementary Table 1. Urinary biomarkers levels according to quartiles of serum creatinine.
(DOCX)

S2 Table Biomarkers levels according to the presence of AKI at admission. (DOCX)

## Abbreviations

AKI: Acute kidney injury
ACLF: Acute-on-Chronic Liver Failure
IL-6: Interleukin 6
MCP-1: Monocyte chemottractant protein 1
OPN: osteopontin
LFABP: Liver-fatty-acid-binding protein
TFF3: Trefoil-factor 3;β2M: β2microglobulin
Cys-C: cystatin-C
MELD: Model of end-stage liver disease
