# Supplementary figures and images for: Urine Monocyte Chemoattractant Protein-1 Is an Independent Predictive Factor of Hospital Readmission and Survival in Cirrhosis
Source: PLoS One. 2016 Jun 30;11(6):e0157371. doi: 10.1371/journal.pone.0157371 (PMC4928797; doi:10.1371/journal.pone.0157371)

## Slide 1
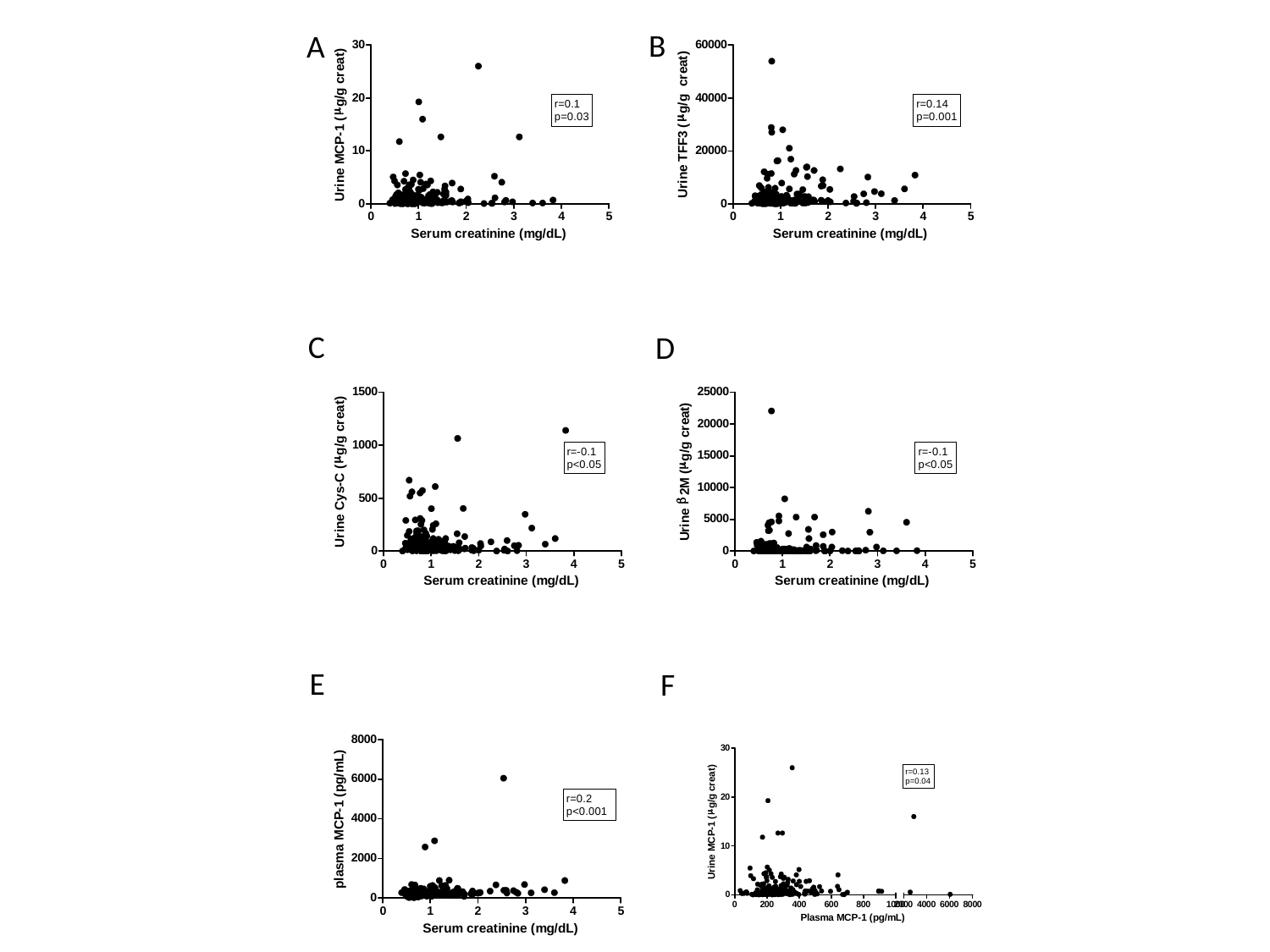

B
A
C
D
E
F

Supplement: S1 Fig — Figure A shows correlation between urine MCP-1 and serum creatinine. Figure B shows correlation between urine TTF-3 and serum creatinine. Figure C shows correlation between urine Cys-C and serum creatinine. Figure D shows correlation between urine β2M and serum creatinine. Figure E shows correlation between plasma MCP-1 and serum creatinine. Figure F shows correlation between urine MCP-1 and plasma MCP-1 levels. MCP-1: Monocyte chemoattractant protein 1, OPN: osteopontin, TFF3: Trefoil-factor3, LFABP: Liver-fatty-acid-binding protein, Cys-C: cystatin C, β2M: β2microglobulin (PPTX) [file pone.0157371.s001.pptx]
