# Supplementary material for: Urine Monocyte Chemoattractant Protein-1 Is an Independent Predictive Factor of Hospital Readmission and Survival in Cirrhosis
Source: PLoS One. 2016 Jun 30;11(6):e0157371. doi: 10.1371/journal.pone.0157371 (PMC4928797; doi:10.1371/journal.pone.0157371)
Supplement: S2 Fig — (PPTX) [file pone.0157371.s002.pptx]

## Slide 1
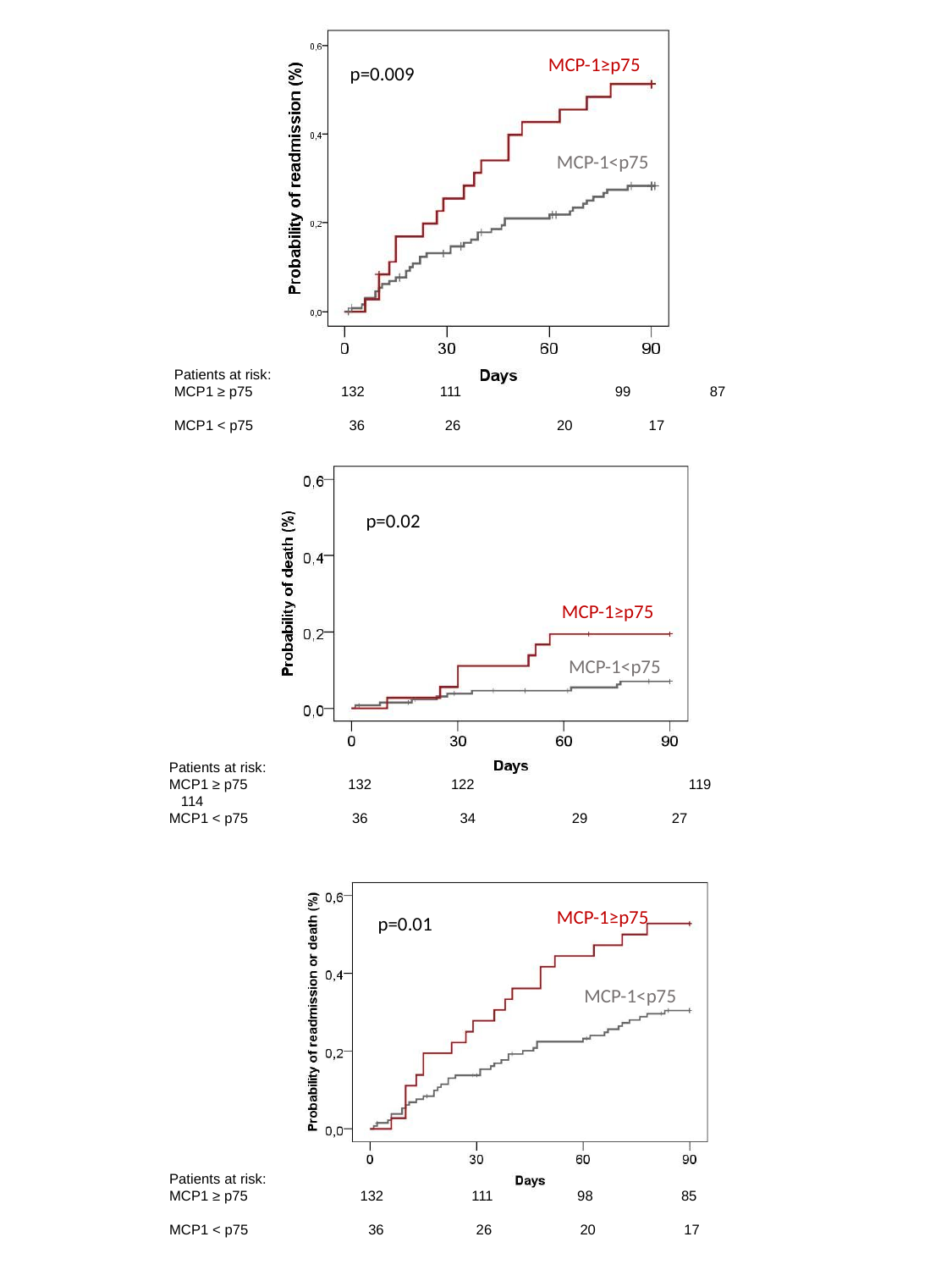

MCP-1≥p75
p=0.009
MCP-1<p75
Patients at risk:
MCP1 ≥ p75 	 132	 111 	 99	 87
MCP1 < p75	 36 26 20 17
p=0.02
MCP-1≥p75
MCP-1<p75
Patients at risk:
MCP1 ≥ p75 	 132	 122		 119	 114
MCP1 < p75	 36 34 29 27
MCP-1≥p75
p=0.01
MCP-1<p75
Patients at risk:
MCP1 ≥ p75 132 111 98 85
MCP1 < p75	 36 26 20 17
