# Supplementary material for: Urine Monocyte Chemoattractant Protein-1 Is an Independent Predictive Factor of Hospital Readmission and Survival in Cirrhosis
Source: PLoS One. 2016 Jun 30;11(6):e0157371. doi: 10.1371/journal.pone.0157371 (PMC4928797; doi:10.1371/journal.pone.0157371)
Supplement: S1 Table — Urinary biomarkers levels according to quartiles of serum creatinine. (DOCX) [file pone.0157371.s003.docx]

**Supplementary Table 1. Urinary biomarkers levels according to quartiles of serum creatinine.**

|  | **Q1**  (n=57) | **Q2**  (n=52) | **Q3**  (n=55) | **Q4**  (n=55) | **p**  **value** |
| --- | --- | --- | --- | --- | --- |
| Cr range^*^ | 0.4-0.7 | 0.71-0.86 | 0.87-1.24 | 1.25-3.84 | --- |
| MCP-1^*^ | 0.43 (0.17-0.85) | 0.49 (0.2-1.1) | 0.85 (0.3-1.5) | 0.57 (0.2-1.9) | 0.07 |
| OPN ^*^ | 1043 (544-3241) | 1760 (702-4053) | 1188 (438-4006) | 1808 (556-3552) | 0.7 |
| LFABP^*^ | 20 (9-51) | 30 (12-50) | 18 (5-32) | 16 (7-35) | 0.2 |
| TTF-3^*^ | 720 (366-1686) | 1019 (380-3607) | 1040 (487-2589) | 1512 (672-5522) | 0.02 |
| Albumin^*^ | 7.5 (27-108) | 5.9 (1.3-22.5) | 6.3 (2-38) | 14 (4-30) | 0.3 |
| Cyst C^*^ | 47 (28-108) | 47 (19-95) | 24 (12-63) | 26 (7-80) | 0.03 |
| B2M^*^ | 113 (54-305) | 113 (41-432) | 67 (11-222) | 67 (11-632) | 0.03 |

Cr range: range of creatinine, expressed as mg/dL.

Data are expressed as median (interquartile range). *All biomarkers are expressed as µg/g creatinine except for albumin which is expressed as mg/g creatinine.

MCP-1: Monocyte chemotactic protein 1, OPN: osteopontin, LFABP: Liver-fatty-acid-binding protein, TFF3: Trefoil-factor 3, Cys-C: Cystatin-C, β2M: β2Microglobulin.
