# Supplementary material for: Urine Monocyte Chemoattractant Protein-1 Is an Independent Predictive Factor of Hospital Readmission and Survival in Cirrhosis
Source: PLoS One. 2016 Jun 30;11(6):e0157371. doi: 10.1371/journal.pone.0157371 (PMC4928797; doi:10.1371/journal.pone.0157371)
Supplement: S2 Table — (DOCX) [file pone.0157371.s004.docx]

**Supplementary Table 2. Biomarkers levels according to the presence of AKI at admission.**

|  | **AKI^*^**  **(n = 50)** | **No AKI**  **n=(168)** | | **p** |
| --- | --- | --- | --- | --- |
| Urine biomarkers: |  |  | |  |
| MCP-1 * | 0.66 (0.25-2.77) | 0.50 (0.21-1.2) | | 0.12 |
| OPN* | 2085 (719-3909) | 1267 (513-3268) | | 0.07 |
| Albumin** | 14 (4-37) | 7 (2-27) | | 0.07 |
| LFABP* | 20 (9-46) | 20 (8-46) | | 0.5 |
| TFF-3* | 2428 (935-2428) | 810 (390-2534) | | <0.001 |
| β2M* | 86 (25-637) | 90 (25-231) | | 0.6 |
| Cys-C* | 31 (13-102) | 37 (14-79) | 0.8 | |
| Plasma MCP-1^#^ | 310 (220-452) | 239 (187-306) | <0.001 | |

Data are expressed as mean±SD, median (interquartile range). MCP-1: Monocyte chemoattractant protein 1, OPN: osteopontin, TFF3: Trefoil-factor3, LFABP: Liver-fatty-acid-binding protein, Cys-C: cystatin C, β2M: β2microglobulin. *Expressed as µg/g creat. **Expressed as mg/g creat. # Expressed as pg/mL.
